# A Great Medicinal Recipe

**Published:** 1855-05

**Authors:** 


### A GREAT MEDICINAL RECIPE.

A new use for physic, not to kill men, nor rats nor bed-bugs, but to detect counterfeit bank notes. The daguerreotype principle has been so much improved, that not only a facsimile of a man's face can be imprinted on a metallic plate, but the facsimile of any bank note can be imprinted on bank note paper, so that no bank teller can by the most minute examination say which is the counterfeit or which the genuine; and scientific men have supposed, that therefore, the days of bank notes were ended; but they reckoned without the doctor. Half a dozen grains of corrosive sublimate, dissolved in a teaspoonful of water, will kill a man, and a million of bed bugs; but if it is spread over a daguerreotyped bank note, with a camel's hair pencil, it instantly obliterates the letters, while it has no effect on a common printed note.

As the time may come when every man who handles paper money, will have to keep by him a small bottle of the solution above mentioned, and might happen in the hurry of business to associate it in his mind with other bottles, and drink it down in a fit of absence of mind, it is well enough to add, that as the man would be dead before the doctor could come, the white of an egg or two beat up in water and swallowed down instantly, will be an antidote. If eggs are not at hand, the next most accessible expedient is to swallow a table spoon or two of flour, stirred quickly in a glass of cold water, or drink largely of fresh milk; ten minutes delay is death. But in any event send for a physician.

---

How True.—"The forms and ceremonies of politeness may be dispensed with, in a measure, in the relations and intimacies of one's own fireside, but kind attentions never."

